# Atropine

**Published:** 1910-01

**Authors:** H. H. Redfield

**Affiliations:** Professor of Therapeutics, Illinois Medical College; Department of Medicine, Loyola University, Chicago, Illinois


### TEXAS MEDICAL JOURNAL.

Established July, 1885

F. E. DANIEL, M. D., - - - - - - Editor, Publisher and Proprietor

Published Monthly.—Subscription $1.00 a Year.

VOL. XXV. AUSTIN, JANUARY, 1910. No. 7

The publisher is not responsible for the views of the contributors.

### Original Articles.

For Texas Medical Journal.

### Atropine.

BY H. H. REDFIELD, M. D.,

Professor of Therapeutics, Illinois Medical College; Department of Medicine, Loyola University, Chicago, Illinois.

The official alkaloid of *Atropa belladonna,* common name deadly nightshade, a European plant of the natural order of *Solanaceae,* and which contains in addition to the official alkaloid atropine, the alkaloids belladonnine, hyoscine, hyoscyamine, and atropine, also a coloring principle called atrosin. Dose of the standard granule of atropine, gr. 1-250.

PHYSIOLOGICAL ACTION.

Atropine has been classed as an irritant, narcotic, mydriatic, antispasmodic, and anodyne. In small doses it produces stimulation of the cardiac apparatus as well as the respiratory and exerts a stimulating effect upon the spinal cord. In large doses it paralyzes the endings of the secretory nerves, and stimulates the sympathetic system.

Applied locally, atropine diminishes the sensibility of the sensory nerves, and when absorbed it produces systemic effects.

Administered internally it checks the secretions of the mouth, nose, throat and larynx, thereby producing a dryness of the mucous surfaces due to the paralyzing action of the drug on the peripheral nerve endings. At first it lessens the gastric and intestinal secretions, but its later action is to produce a profuse secretion from the gastric and intestinal mucous membranes.

Under atropine the heart is at first slowed, but later the heart action becomes very rapid and the force is augmented, the pulse grows exceedingly rapid, arterial tension is high and the circulation is increased. This action of atropine is brought about by its stimulating effect on the cardio-sympathetic ganglia, and its paralyzing action upon the intra-cardiac ganglia, thus producing a stimulation of the accelerator apparatus, while lessening inhibition. It differs from digitalin inasmuch as that drug increases both.

The entire vasomotor system is stimulated at first, but afterwards by over-stimulation it is paralyzed. The beat and force of the heart grow weaker, the blood vessels relax and lose their tone, with the result that blood pressure undergoes a marked fall. There is complete motor paralysis, delirium, coma, and finally death due to asphyxia. This when there has been lethal doses taken.

The exhibition of atropine either locally or internally causes dilation of the pupils, which is produced by stimulating the end organs of the sympathetic and paralyzing those of the motor-oculi, which has the effect of decreasing the influence of the circular fibres, while at the same time it increases the power of the radiating fibres of the iris. Atropine also has the power of paralyzing accommodation and lessening intra-ocular pressure. According to Donders, the smallest quantity of atropine that will produce any effect upon the pupil, is one seven hundred thousandth of a grain (1-700,000).

Large doses of atropine produce cerebral congestion, characterized by a busy delirium, which is frequently accompanied by spectral illusions.

The region of the spinal cord between the second cervical and tenth dorsal vertebrae is stimulated, producing thereby a paralysis of the motor-nerves, central and peripheral; power in the lower extremities being lost first.

There is a slight impairment of sensibility, but the irritability of the muscles is not affected. The respiration is increased, and there is a rise of temperature.

Sometimes the exhibition of atropine is followed by a diffuse scarlet eruption on the skin and fauces, which greatly resembles that of scarlet fever; there is great difficulty in swallowing and the throat is sore. This effect is due to the congestion of the capillaries, and the consequent greatly increased circulation.

THERAPEUTICS.

Persons of a bilious, lymphatic, or plethoric disposition, women and children of a delicate skin; those who are happy, jovial and good entertainers when feeling well, but who become peevish, violent, delirious, and subject to convulsions when indisposed, seem to be especially susceptible to the action of atropine.

They take cold easily and are especially sensitive to drafts of cold air; men who take cold after having their hair cut. There is generally headache of a throbbing nature. The pains come on suddenly, remain only a short time, and leave as suddenly as they came without any apparent cause whatever. The face is red and swollen, eyes congested and injected, pupils dilated and staring.

The carotids pulsate and the pulse is full and bounding, with the mucous membranes of the throat, nose and mouth hot and dry.

Atropine should be studied in all motor disturbances, when there is a condition of anaesthesia, or owing to the great irritability of all the senses there is present marked hyperaesthesia. The muscles jerk or twitch, conditions often met with in puerperal convulsions, the spasms of infancy, epilepsy, hydrophobia, chorea, whooping-cough and locomotor ataxia.

Atropine occupies an honorable place in those mental disorders characterized by marked alternations in the moral tone of the individual—hallucinations, melancholia and rage with illusions of a spectral nature.

In the congestive delirium which frequently attends the acute fevers, or which is due as a result of metastasis to the brain, atropine is the remedy of choice.

Cases of congestive vertigo are relieved by atropine. Congestive, neuralgic or nervous headaches, when the face is flushed, eyes injected, ptosis, head feels as though it were on fire, or the patient may complain of a sensation of blindness, or again there may be flashes of light which dart back and forth across the eyes. The headache is made worse by light, noise, movement or lying down.

The place of atropine in the treatment of arterial congestions of the brain has been firmly fixed.

In the hyperemia of sunstroke it has but one peer, and that is glonoin. The congestions met in cases of injury to the head, when there are all the evidences of concussion present, congestions brought on by great mental excitement excessive indulgence in alcoholic beverages, are promptly met by atropine.

It should be studied in neuralgia which is of recent origin, or which occurs in young patients, especially when associated with hyperaemia and hyperaesthesia. The pains are generally most pronounced about 5 p. m., coming and going quickly, and are made worse by motion. In these cases it will be found as a general rule that the trigeminus is the nerve most affected.

In iritis and other conditions of the eye it gives excellent results.

The dryness, soreness, painful deglutition, swelling and burning sensation, seen in pharyngitis and chronic tonsilitis, are indications which call for the exhibition of atropine.

In all congestions and inflammations of the uterus, when the patient complains of a violent burning sensation with nettle-like pains, and a feeling of fullness as though the uterus were pressing down toward the vulva, atropine should be studied.

Its position in the treatment of fevers is between aconitine and arsenic. The type of fever is usually continuous. The face is red and congested, eyes bright and glistening, and the other general indications for the exhibition of atropine are present. In puerperal fever the exhibition of atropine is imperative, and at the onset of scarlet fever or measles, when the skin is hot and glistening. In the Sydenham type of scarlatina, atropine is said to have a modifying influence on the course of the disease.

It has been used to good effect in peritonitis, when the transverse colon can be felt like a pad. There is soreness in the entire abdominal region, and the tenderness is so great that even the jar of a person walking across the room disturbs the patient.

A dry cough with the sensation of a feather in the larynx, worse at night, calls for atropine; although cicutine should be remembered in these cases.

In pleuro-pneumonia, after bryonin has completed its task and there remains an extreme and disagreeable soreness in the side, atropine will be found effective.

In mastitis when the gland is hot and red, hard and heavy, the face flushed, eyes injected, pulse full and bounding, head throbs and there is an intolerance to light.

